# A cross-sectional analysis of fingerstick blood self-microcollection for remote HIV suppression monitoring in Atlanta, Georgia, USA: a path to expanding access to continuum of care

**DOI:** 10.1186/s12981-025-00781-1

**Published:** 2025-08-30

**Authors:** Jeffrey A. Johnson, Amanda J. Smith, Ruth Dana, Erin Rogers, M. Leanne Ward, Ariana S. Tino, Vickie Sullivan, Wei Luo, Jin-fen Li, Patrick S. Sullivan

**Affiliations:** 1 Division of HIV Prevention Laboratory Branch, Centers for Disease Control and Prevention, 1600 Clifton Rd, NE, MS 18-2, Atlanta, GA 30329 USA; 2 Rollins School of Public Health, Emory University, Atlanta, GA 30322 USA

**Keywords:** HIV viral suppression monitoring, Self-collection, Fingerstick blood, Decentralized testing, Remote care, Continuum of HIV care

## Abstract

**Background:**

Many people with HIV experience considerable barriers to accessing HIV clinic services. Options that would permit blood sampling that preclude the need for in-clinic visits and increase privacy would aid in overcoming many of the obstacles that hinder receiving adequate HIV care.

**Methods:**

In Project Home-MaDE, 57 participants were evaluated for their ability to collect fingerstick blood (minimum 250 µL) in Microtainer tubes (MCT), then package and overnight mail specimens following kit instructions without assistance. Specimens were required to arrive at the laboratory within four days of collection. Plasma viral loads obtained from mailed blood were compared to matched venipuncture samples collected on the same day. For fingerstick-derived plasma, the limit of quantitation was 210 copies/mL, a benchmark relevant for Undetectable = Untransmissible prevention. A non-reactive or below-quantifiable result reflected viral suppression. Self-collected dried blood spots which have historically been used for remote blood sampling were likewise evaluated.

**Results:**

Forty-seven (82%) participants had acceptable MCT samples for testing. Ten specimens were rejected either for excessive time and temperature (*n* = 1) or insufficient sample volume (*n* = 9). Of the 34 participants who initially experienced difficulty in obtaining sufficient sample 29 elected to retry and 24 were successful. All 46 acceptable MCT plasmas tested provided accurate results as compared to the suppression levels in their matched conventional venipuncture viral loads.

**Conclusion:**

Under a rigorous protocol, plasma from mailed, self-collected fingersticks by untrained individuals were suitable for remote viral suppression monitoring. This evaluation, however, was limited to temperatures and courier service in the U.S. Approved testing options for self-collected samples may support HIV telemedicine and empower persons to overcome barriers to care services.

**Supplementary Information:**

The online version contains supplementary material available at 10.1186/s12981-025-00781-1.

## Introduction

Persons with HIV (PWH) in the United States experience barriers to accessing HIV care, including inability to take time away from work, cost of services, stigma and, particularly in rural areas, a limited availability of care providers [1–3]. Impediments to retention in HIV care along the care continuum continue to undermine both successful viral suppression for persons with HIV and prevention of ongoing transmission. Current viral suppression among PWH in the U.S. is 66% [4]. To realize viral suppression goals in Ending the HIV Epidemic [5] and achieve the “Undetectable = Untransmissible” (U = U) aim for PWH, new clinical monitoring tests are needed to reach more broadly PWH. Expanding testing capacity will be contingent on the availability of test procedures that can be performed in settings where persons with HIV are more comfortable. For example, places, residences, or non-clinical settings that are available at all hours of the day and do not require provider oversight.

The absence of HIV nucleic acid detection tests that are approved by the Food and Drug Administration (FDA) for use with self-collected specimens is a barrier to serving persons unable to access conventional care. Suitable self-collected specimens for HIV testing have pre-analytic concerns, such as adequate specimen volume, rapidity in specimen collection to prevent clotting, time to specimen processing, exposure to extreme temperatures, and ease with which blood is obtained to ensure integrity of the sample. Additionally, evaluations of nucleic acid tests for viral suppression monitoring must consider the impact of potential cellular DNA carryover on test results. For any self-directed collection procedure, the instructions must be comprehensible to end-users, the sample collection must be easy to perform, and consideration must be given to cost to preserve accessibility to as many people as possible.

There are point-of-care specimen sampling options available, such as plasma separation cards and tubes. However, in our experience the plasma cards do not provide the same level of stability and sensitivity as liquid blood, and plasma separation tubes require centrifugation within 6 h of collection per the package insert. Furthermore, point-of-care viral load platforms are available outside the U.S. and may be an option in the future, but these still require in-person clinic visits. Here we were interested in sample types that allowed for maximum unprocessed handling time, supported plasma testing with little additional sample manipulation, did not require travel to a clinic, and can be performed on platforms that are widely available in the U.S. Hence, we elected to use plasma that could be obtained from K2 EDTA preservative Microtainer tubes (MCT) using fingerstick blood (FSB).

To develop the procedure reported here for self-microcollection of fingerstick blood in MCT, it was necessary to first develop specimen handling and acceptance criteria, then we evaluated participant-collected specimens in a head-to-head comparison with clinic-collected venipuncture blood for the purpose of monitoring viral suppression. We conducted a similar analysis with matched dried blood spots (DBS) as a comparator, due to its extensive use in many countries.

Two primary study endpoints were evaluated: (1) the specimen suitability and (2) the comparability of the MCT and DBS specimens returned. A specimen was suitable if a sufficient volume was collected and had arrived at the laboratory within established transport conditions. Comparability was determined by agreement between the viral load (VL) test results on the venipuncture (gold standard) and the self-collected specimens. Secondary endpoints included rate of return of self-collected specimens, specimen package temperature by season, VL results compared to the U = U benchmark (200 copies/mL), and the percent undetectable among persons who reported being prescribed antiretrovirals (ARVs). Based on these data, we evaluated the suitability of self-collected MCTs that were mailed for VL testing as an option for remote viral suppression monitoring.

## Methods

### Human ethics and consent to participate

In accordance with the Declaration of Helsinki, Project Home-MaDE (At-Home Microtainer and Dried Blood Spot Evaluation) received IRB approval from Emory University on October 19, 2021 (IRB ID: STUDY00003185) and a Centers for Disease Control and Prevention (CDC) project determination approval to begin on October 30, 2021 (project ID 0900f3eb81dccecc). All participants provided written consent to participate. Consent forms and all identifiable data collected were maintained under lock at the study clinic or at the Emory Rollins School of Public Health. All participants were assigned a unique study identifier to identify specimens; no personally identifiable information was provided to the CDC.

The study recruitment goal was 50 participants who were eligible if 18 years of age or older, residing in Atlanta, GA, willing to travel to Emory University for two study visits, willing to have blood drawn by venipuncture, and willing to self-collect MCT and DBS blood samples. Among the 50 participants, we were interested in evaluating VL results from participants with HIV who were not virally suppressed (*n* = 25), and persons with undetectable VL, including persons without HIV (PWOH) and PWH who were fully suppressed on ARVs (*n* = 25).

The primary method of recruitment was through referrals from prior health studies in which participants consented to be recontacted for future research. Participants were identified from prior health study recontact lists as being potentially eligible for the current study. A recruitment email, text message, and/or phone call was sent to participants to introduce the study as well as explain how interested participants were able to join. Eligibility screening was performed by an initial phone interview. Participants were provided $25 for each self-collected and venipuncture sample. The total incentive amount would be $100 for the two venipunctures, one MCT, and one DBS samples. If participants who initially had difficulty collecting sufficient sample desired a recollection, they were provided an additional $25. In later attempts to meet enrollment of unsuppressed PWH, incentives were raised to $50 per blood sample.

### Viral load protocol for plasma derived from fingerstick blood

We used a 1:7 dilution protocol with fingerstick blood for semi-quantitative detection (see detection limits in Results below) of HIV-1 RNA by incorporating the following pre-analytic steps beyond the information in the FDA-approved package insert for the Aptima^®^ HIV-1 Quant Dx assay (package insert ver. AW-18107-001 Rev. 002 April-2021) using the Hologic Panther™ system: (1) allowance for self-collected and healthcare worker-collected fingerstick blood; (2) mailing of specimens at ambient temperatures by patient or healthcare worker for express delivery; (3) expanded time and temperature ranges prior to specimen processing to support specimen transit conditions; and (4) a 1:7 dilution of the plasma fraction prior to analysis. The 1:7 dilution protocol uses the standard automated Panther onboard testing process. The numeric onboard result provided by the assay is multiplied by 7 to yield the reported numeric viral load for each MCT specimen.

The plasma derived from venipunctures were tested following the approved package insert using 700 µL of undiluted plasma. The MCT plasma protocol involved adding 100 µL plasma to an Aptima Specimen Aliquot Tube (SAT), to which 600 µL Aptima diluent is added to obtain a 700 µL specimen volume (1:7 dilution). For both the standard and dilution protocols, 500 µL was the reaction volume used for testing. For all FSB VL measurements we applied an acceptable variance of ± 0.5 log_10_ compared to venipuncture samples to account for the reported assay error for VL tests [6, 7].

### Viral load protocol for DBS

VL testing of DBS was performed on the Abbott m2000 using four 6 mm punches from a blood spot. Prior to removing cryostored DBS from plastic bags for testing, the specimens were thawed at room temperature (18–25 °C) for 30 min. For each participant, the DBS punches were submerged in 1.3 mL DBS buffer in Abbott master mix tubes. The tubes were vortexed and incubated in a heating block at 55 °C for 30 min. Following heating, the tubes were centrifuged at 870 xg for 5 min and then processed using Abbott DBS protocol “1.0 ml HIV-1 RNA IUO US TTv11” with the RealTime HIV-1 test (package insert ver. 51-602146/R6 December 2011). Due to limitations of the DBS specimen volume, the limit of quantification with the DBS protocol is 832 copies/mL. The two different viral load platforms were used because each had validated protocols established for diluted plasma and DBS, respectively.

### Establishing specimen handling and acceptance criteria

Specimen handling limits were first determined from bench evaluation of extensive environmental conditions. To simulate how reference testing of self-collected blood could occur, the MCT evaluation for VL testing was performed following strict Clinical Laboratory Improvement Amendments (CLIA) workflow for specimen accessioning and testing in the CDC HIV Reference Laboratory. Return specimen packaging and pre-analytical inspection at the laboratory assessed specimen integrity. The screwcap MCT specimen tube evaluated contained K2-EDTA (dried) anticoagulant to allow for blood plasma separation and had specimen volume marks for 250 and 500 µL. To obtain 100 µL plasma for the viral load assay, a minimum of 250 µL FSB would be required; this amount of blood volume is equivalent to filling a complete 5-circle DBS card.

Before recruiting participants, we evaluated the analytical performance of 250 µL whole blood specimens in MCTs under different conditions (time, temperature, and virus concentration) to establish acceptable specimen conditions for VL testing. First, we established a baseline performance of the 1:7 dilution protocol by preparing and processing whole blood specimens in the laboratory on the same day. For this evaluation, virus was diluted in 250 µL whole blood in triplicate at concentrations prepared for the 25 °C assessment (see Table 1) and processed at room temperature by centrifugation at 1200 xg for 15 min to separate the plasma. The 1:7 dilution protocol limit of quantitation (LOQ) has been reported as 400 copies/mL if specimen processing and handling occur within 24 h from time of collection [8]; however, additional work described below expands on this limit.

After assessing the same-day performance, other conditions outlined in Table 1 were performed, varying the temperature and virus concentrations to ascertain the maximum allowable conditions under which the results of VL testing were robust. Some experiments were repeated because the diluted virus concentrations did not contain the expected levels; however, those data were included to yield a more thorough dataset. Furthermore, the results from each experiment were used to design the next set of conditions because fresh whole blood was limited and so each evaluation was tailored to the available blood volume at the time. For the purposes of investigating the viral suppression threshold treatment as a prevention benchmark of 200 copies/mL for U = U, we had particular interest in the ability to discern VL results around this benchmark (2.3 log_10_). In addition to assessments of virus concentration and temperature, lysed OM10.1 cells [9] containing proviral DNA were spiked into whole blood in MCTs and were tested with the 1:7 viral load protocol to assess any impact of lysed cells on VL results.

**Table 1:** Conditions evaluated for detection of HIV viral load in 250 µL whole blood specimens in MCTs spiked with HIV-1 at the indicated concentrations

Constant Temperature (^o^C)	Time (hours)	Log_10_ viral load concentrations (copy/mL) prepared
2	0, 120	4.54
25	0, 120	2.28^a^, 2.54, 2.60, 3.00, 3.74
30	0, 24	2.54
32	0, 24, 48, 96	3.50
35	0, 120	2.28, 2.70, 3.30, 3.54, 4.54, 6.60, 7.84
37	0, 120	2.28, 2.54, 3.0, 3.60, 3.74
38	0, 24, 48	2.24 ^b^, 3.54
39	0, 96, 168	3.50
40	0, 18	2.24 ^b^, 3.54
46	0, 8	2.24 ^b^, 3.54

Equivalent to ^a^ 190 and ^b^ 175 copies/mL which are below the limit of quantitation (LOQ)

### Preparation and instructions for MCT kits

The specimen collection kits contained either K2EDTA preservative Microtainer tubes (MCT, Becton-Dickinson, SKU 365974) or dried blood spot cards (DBS, Whatman 903 Protein Saver SKU 10534612) together with a high flow contact-activated lancet (2 mm depth, Becton-Dickinson, SKU 366594). Participants were given pictographic instructions providing optimal techniques for specimen collection, packaging and mailing procedures (see Supplementary Figure S1). The instructions were revised following feedback by end-users on steps that needed clarification. In addition to the infographic provided in the collection kit, two-minute instructional videos were created and are accessible on YouTube (for MCT collection: https://www.youtube.com/watch?v=dxzEFRQrctg, and for DBS collection: https://www.youtube.com/watch?v=WvXQX60KIC8).

Participants were asked to collect and ship two self-collected samples: one MCT (visit 1) and one DBS card (visit 2), with the specimens collected two weeks apart. For the MCT collection, the participant was provided an MCT collection and mail-return kit and was instructed to go to a private room in the research clinic that simulated a home environment to perform the fingerstick collection. The participant was unsupervised during the collection and had only the provided instruction material as a guide (See Figure S1). After completing the FSB collection per instructions, participants packed the specimen for drop off at a FedEx store or box location of their choice within 24 h after leaving the clinic. Prior to leaving the clinic on the day of MCT collection, a phlebotomist collected a venipuncture blood sample in EDTA that was processed as a gold-standard comparator to the self-collected FSB.

The MCT specimen bags used for the shipped specimens had an irreversible temperature sticker (Cole-Parmer, SKU EW-08068-20) that recorded the maximum temperature to which the specimen was exposed during transit. For the DBS self-collection, the participant was mailed the DBS kit to perform at home. The DBS specimen bags contained desiccant packs to maintain humidity < 50% and a humidity indicator card. All self-collected specimens were shipped at ambient temperature to the CDC HIV laboratory for evaluation as described below.

### Specimen receipt and condition evaluation

Specimens received at the CDC laboratory were accessioned and examined for adequate specimen volume, temperature reading, and days since collection. For MCTs, the temperature indicated on label affixed to the specimen bag was recorded and specimens that had reached 37 °C or above during shipment were rejected. MCTs with FSB volumes of > 200 µL were accepted for an assessment of recoverable plasma volume. Samples that yielded ≥ 100 µL plasma were included in the comparison between VL results from MCTs and venipuncture blood. FSB samples of ≥ 500 µL would provide sufficient plasma for repeat testing if desired. Any sample providing insufficient plasma was rejected and recorded as quantity not sufficient. In addition to plasma volume, the hemolysis index (see Supplemental Figure S2) of plasma was noted as a proxy indicator of difficulty in obtaining the FSB. An index above 2 + was presumed to be a difficult collection.

DBS received at the CDC laboratory were inspected for completeness of blood spots and the percent humidity displayed on the enclosed indicator card was recorded. Nucleic acids in DBS are subject to hydrolytic degradation so humidity > 50% may affect sample integrity. Samples without complete blood circles were rejected for testing. After inspection, DBS cards were packed in plastic bags with new desiccants and humidity indicator card. The DBS cards were then stored at -20 °C on the same day received until VL testing.

### Evaluating correlation and agreement between venipuncture and self-collected blood viral suppression results

Correlation between the viral suppression interpretations obtained from conventional clinic-drawn venipuncture viral load testing and testing of self-collected specimens mailed in by participants was measured by the Pearson’s correlation coefficient (*r*). The agreement between venipuncture and self-collected fingerstick blood in their ability to measure when suppression is above or below the EHE benchmark of 200 copies/mL was assesses by the Kappa statistic (Κ).

## Results

### Analytic performance with simulated environmental conditions

#### Quantitative metrics for MCT prepared in laboratory and processed onsite within 24 h

We first assessed performance of the 1:7 dilution protocol for the least challenging conditions, that being a specimen collected and processed onsite the same day. All viral load comparisons reported are relative to the actual quantitated results at baseline (Day 0) for the virus dilutions prepared. Table 2 provides for the interpretation of 1:7 dilution protocol results for virus in whole blood under same-day testing conditions. Specimens with results listed as “Not Detected” have no detectable HIV-1 RNA. Specimens with onboard results listed as “<30 detected” (< 210 final concentration) are detectable but unquantifiable. Specimens with results ≥ 210 copies/mL to 70,000,000 have detectable HIV-1 RNA that is quantifiable. The final result reported (as copies/mL) is after multiplying the onboard result values by a factor of 7 to adjust for the plasma dilution and is shown in the shaded column.

**Table 2:**
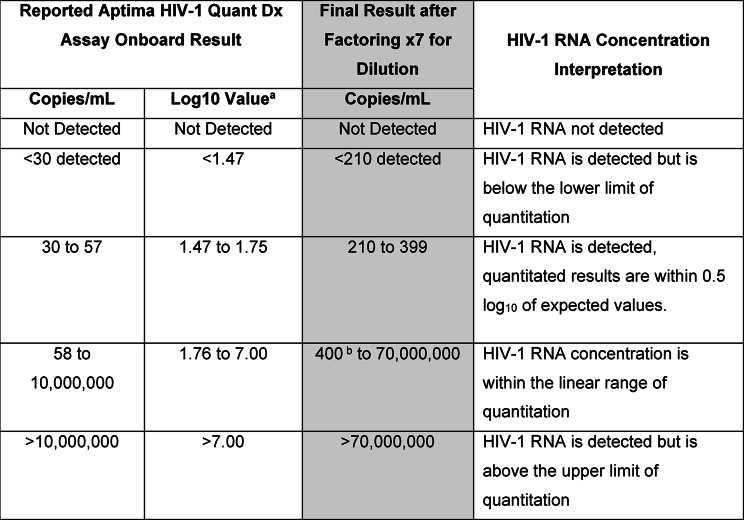
Onboard result interpretation of the 1:7 Dilution protocol for on-site collection and plasma processing within 24 h

^a^Values are recorded to two decimal places

^b^Lower limit of quantification published in reference 6

**Table 3:** Viral load results for plasma obtained from 250 µL whole blood in MCTs spiked with HIV-1 at the concentrations and conditions indicated in Table 1. Contrived venipuncture blood specimens were tested in triplicate. For FSB one sample per condition was tested

Constant Temperature (^o^C)	Time (hours)	Measured log_10_ virus concentrations^a^						
2	0	4.54						
	120	4.47 (4.53, 4.44)						
25	0	< 2.32 ^b^	2.51	2.58	2.93	3.70		
	120	< 2.32 ^b^ (2.35, < 2.32)	2.47 (2.58, 2.36)	2.49 (2.64, 2.39)	2.85 (2.88, 2.83)	3.67 (3.73, 3.62)		
30	0	2.45 (2.52, < 2.32)						
	24	2.40 (2.57, < 2.32)						
32	0	3.52						
	24	3.39						
	48	3.55						
	96	3.21						
35*	0	< 2.32 (2.40, < 2.32)	2.70 (2.81, 2.52)	3.16 (3.29, 3.06)	3.54	4.54	6.60	7.84
	120	< 2.32	2.40 (2.47, 2.24)	2.89 (2.98, 2.73)	3.18 (3.29, 3.09)	4.16 (4.15, 4.17)	6.66 (6.77, 6.43)	7.53
37	0	< 2.32	2.51	2.93	3.22	3.70		
	120	< 2.32	2.33 (2.41, < 2.32)	2.56 (2.62, 2.41)	2.93 (3.02, 2.87)	3.40 (3.47, 3.32)		
38	0	< 2.32	3.59 (3.68, 3.48)					
	24	< 2.32	3.36 (3.42, 3.30)					
	48	< 2.32	3.38 (3.43, 3.35)					
39	0	3.50						
	96	< 2.32						
	168	Not detected						
40	0	< 2.32	3.59 (3.68, 3.48)					
	18	< 2.32	3.47 (3.51, 3.43)					
46	0	< 2.32	3.59 (3.68, 3.48)					
	8	< 2.32	3.37 (3.45, 3.31)					

This table includes more VL concentrations than described in Table 1 as dilution errors were also included. “0” hours are the actual VLs measured at baselines. *Calibrated temperature read 36 °C for two of the five days; ^a^ for venipuncture whole blood testing in triplicate the mean (upper, lower) concentrations are indicated; ^b^ <2.32 signifies detected but below the assay limit of quantitation

#### Quantitative evaluation of MCT samples to establish the most extreme acceptable environmental conditions

Viral load results for MCT specimen conditions listed in Table 1 are provided in Table 3. All incubation periods up to 35/36 °C resulted in no substantive loss of VL compared to baseline. After five days of constant 37 °C there was observed a nominal loss of virus concentration; however, these decreases were less than 0.5log_10_ copies/mL (additional data on this condition are provided below). Higher temperature conditions were evaluated at 3,500 copies/mL and close to the lower limit of RNA detection for diluted MCT specimens to assess the robustness of low-copy specimens. At 25 copies/100 µL of MCT-derived plasma, below what is quantifiable for the dilution protocol, virus was still detectable in each of the triplicate aliquots after 48 h at 38 °C. At 46 °C, the most extreme temperature evaluated, virus detection was uniformly maintained after 8 h. Moreover, lysed OM10.1 cells in whole blood did not yield any detectable virus with the HIV-1 Quant Dx assay at an equivalence of 1000 copies HIV DNA/mL whole blood, which reflects the range observed in persons with associated plasma viremia below 200 copies/mL [10].

**Fig. 1:**
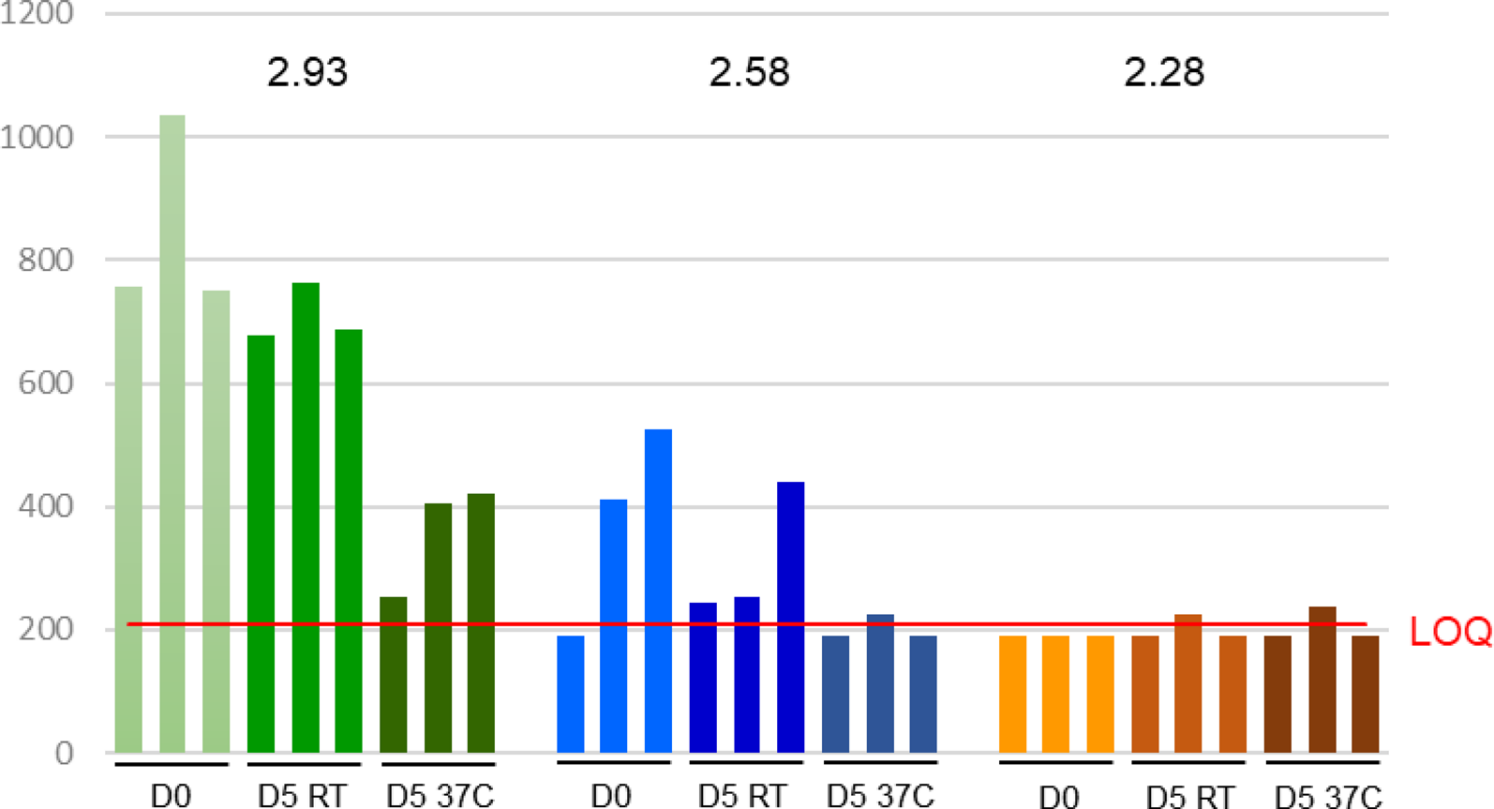
Variability in low copy virus load detection, in triplicate, of HIV in MCT whole blood at baseline (D0) and after 5 days at constant room temperature (RT, 25 °C) or at 37 °C. The numbers above the bars are the mean log_10_ quantitated concentration at baseline for each group. The red horizontal line is the limit of quantitation U = U suppression benchmark. Virus detected at less than the limit of quantification (LOQ, < 2.32 log_10_) are assigned as 2.28 log_10_ for illustrative purposes

**Fig. 2:**
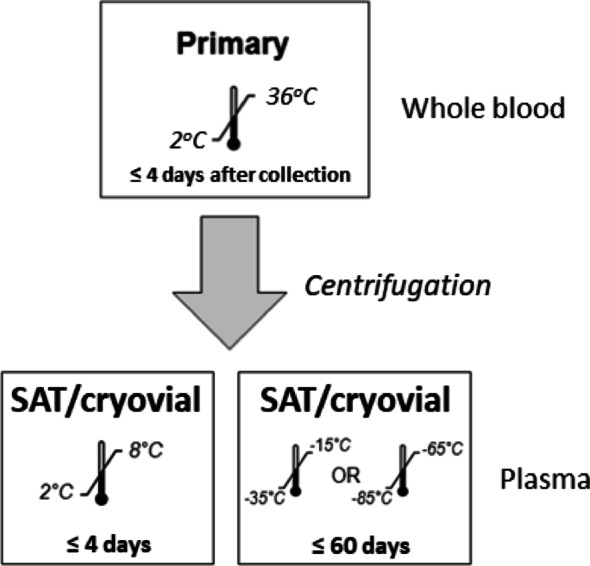
Microtainer (MCT) specimen transport and storage conditions. After centrifugation of fingerstick whole blood the plasma fraction can be stored short-term at 4 °C or frozen for up to 60 days prior to testing

Table 4 depicts the changes in RNA concentrations under sustained 37 °C temperature for 5 days. An example of the variability observed at quantitating low virus copies after five days at room temperature (25 °C) and 37 °C is illustrated in Fig. 1. Under the stressed environment of five consistent days at 37 °C we observed with the 1:7 MCT plasma dilution protocol that HIV-1 RNA concentrations of 250 to 600 copies/mL at collection (baseline) resulted in HIV-1 detected and quantitated or HIV-1 was detected but below the lower limit of quantitation. Concentrations of > 600 to 70,000,000 copies/mL at baseline result in HIV-1 detected and quantitated. Thus, a constant 37 °C for 5 days is the condition under which specimens with VL of < 600 copies/mL might not always be discernable from specimens with detectable virus that is below the 200 copies/mL benchmark. While higher temperatures are tolerable for shorter periods (see Table 2), to allow a broader window for specimen transport we selected a slightly lower temperature. Therefore, we established the maximum tolerance for the self-collection evaluation at a slightly reduced condition of four (4) days and 36 °C/96.8 °F (Fig. 2).

### Field evaluation of the self-collection method

#### Participant recruitment

PWOH or PWH but known to be suppressed on ART were able to complete enrollment over a period of 9 months. PWH but unsuppressed were recruited at a much slower rate as the level of participant engagement was much lower. This prompted the enrollment period to be extended to a total of 24 months to attempt to enroll 25 unsuppressed persons and involved referral recruitment of PWH from local community-based organizations and clinics. However, we could not obtain demographic data on those enrollees as they hadn’t been in prior projects, and we didn’t employ a survey instrument for this project. Furthermore, we added transportation assistance by arranging taxi service for appointments to lower access barriers and also offered appointments on evenings and weekends. For both groups the enrollment period included recontact and additional collection attempts if their specimens were rejected as detailed in the next section. Enrollment was ultimately halted due to futility in the inability to recruit additional PWH who were not virologically suppressed.

**Table 4:**
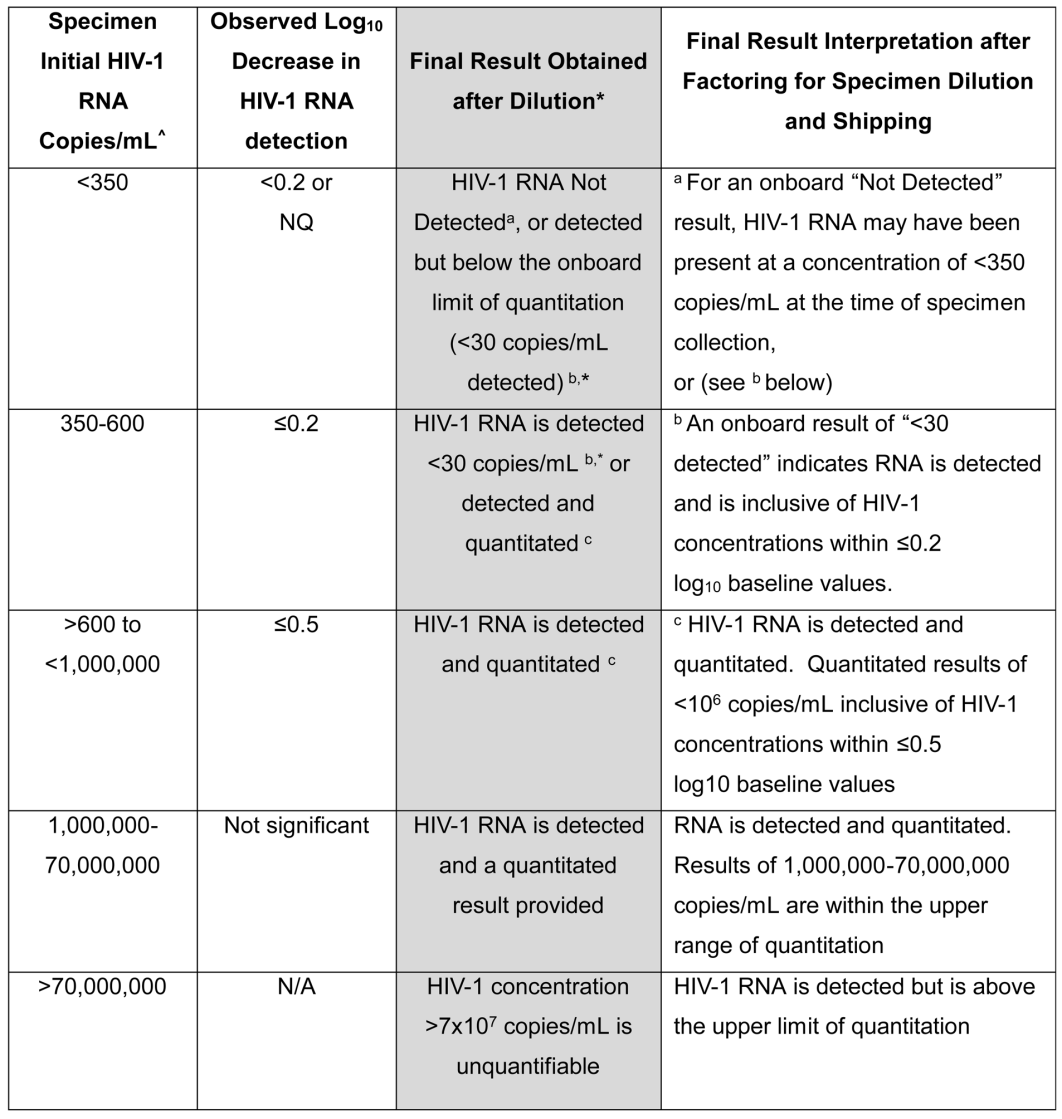
Observations and final interpretation of FSB results at 37 °C for 5 days factoring for the 1:7 Dilution

^^^Specimen volume of 250 µL. NQ, not quantifiable. *See Table 2 for onboard limit of quantification

#### Provision of acceptable self-collected MCT specimens

**Fig. 3:**
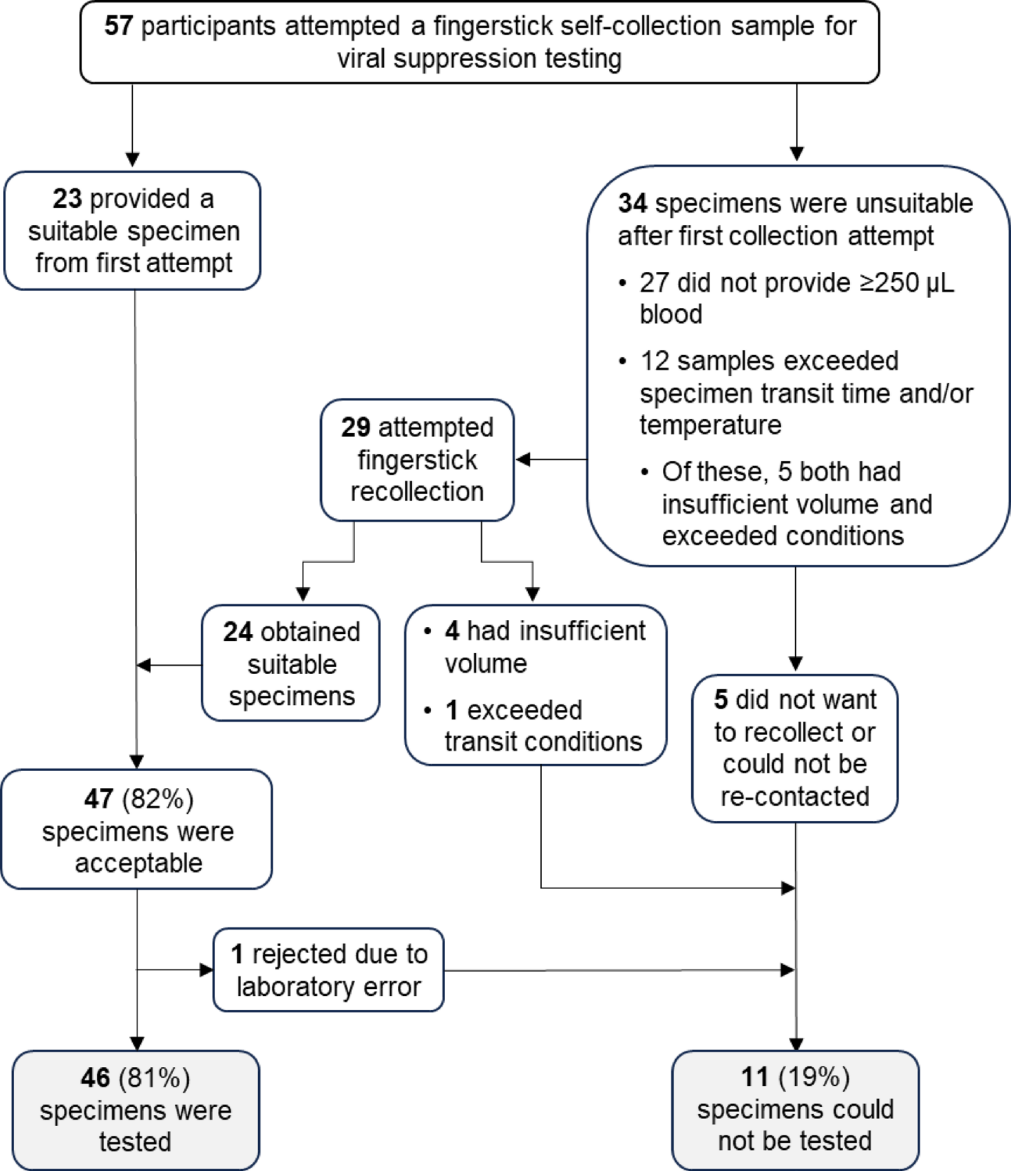
Flow diagram of specimens accepted and rejected from participant MCT self-collections

**Fig. 4:**
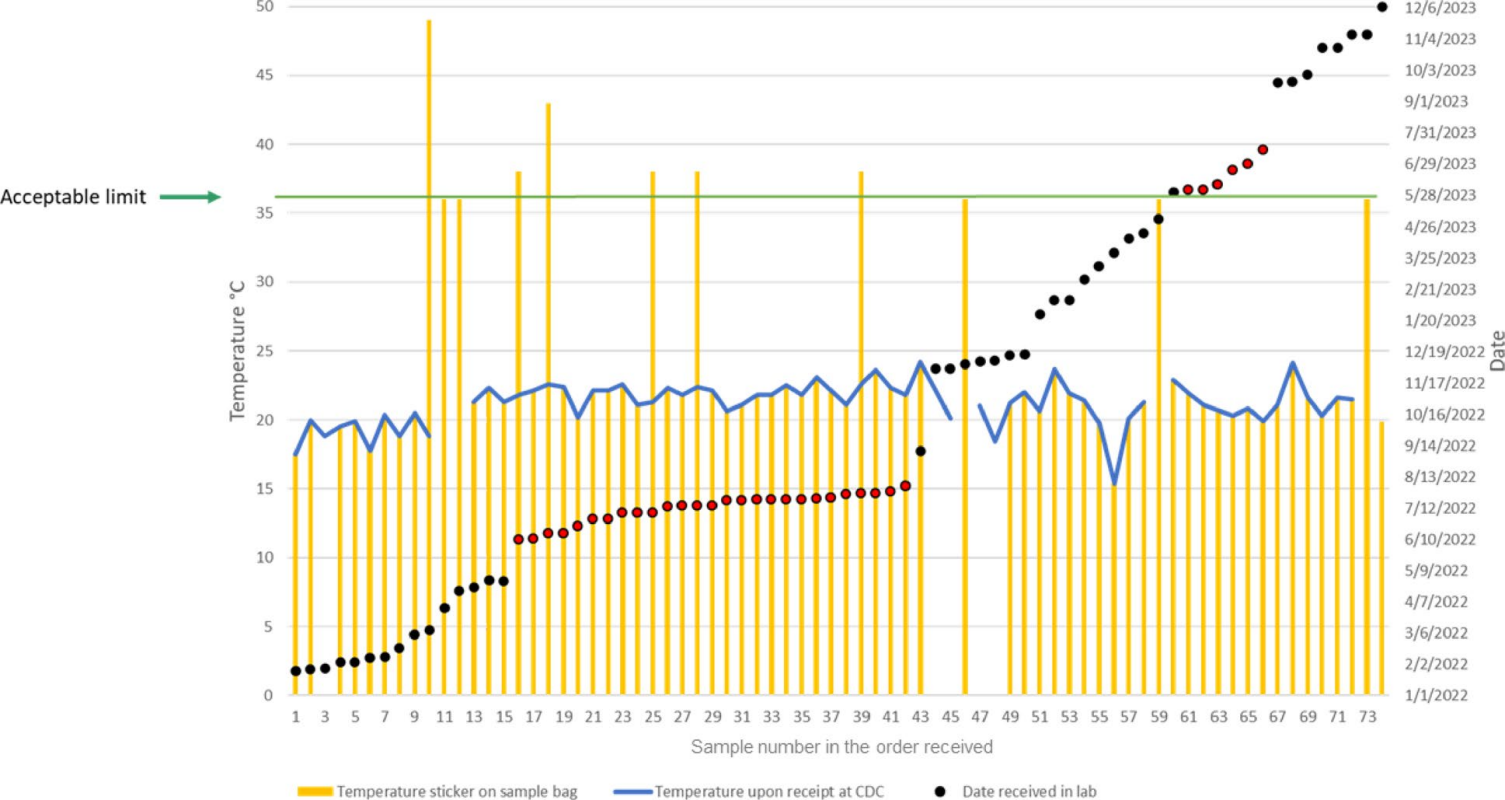
Recorded temperatures of MCT specimens mailed by participants. The irreversible temperature labels on specimen bags (yellow bars) do not record temperature below 37 °C, therefore, for those the package temperatures recorded by CDC specimen receiving (blue line) is the temperature of record. Specimens received during summer months are depicted as red dots. Specimens without a temperature sticker are not shown and were rejected

Laboratory acceptance of MCT specimens followed the criteria outlined in the Methods. Of 57 enrollees attempting their first MCT self-collection, 34 participants at first attempt either did not provide sufficient quantity of FSB (*n* = 27) or exceeded the allowable specimen transit time and/or temperature (*n* = 12) (Fig. 3). In examining the specimen temperature labels of all specimens received, we found five samples between the summer season (May-August) had exceeded 36 °C during transit; however, one sample that also had experienced the highest temperature (49 °C) had arrived outside of the summer months (Fig. 4). Samples from five individuals had both insufficient quantity and exceeded allowable transit conditions. Of the 34 participants with initially unacceptable specimens, 29 (85%) opted to perform an additional collection of whom all but four were able to collect a suitable volume; however, one specimen was rejected due to exceeding both the allowable time and temperature. Of the participants with adequate FSB, 77% of specimens were received at the laboratory within two days, and 50% provided a sample volume of ≥ 500 µL. Five participants either did not wish to attempt a further recollect or we were not able to re-contact. Thus, 10 participants provided insufficient specimens. The resulting 47 specimens were processed for testing, however, a laboratory specimen accessioning error caused one more specimen to be rejected under the strict accessioning criteria. Ultimately, 46 participant samples were tested per protocol, which included 11 PWH not virologically suppressed, 22 PWH who were virally suppressed, and 13 PWOH.

In assessing hemolysis index of all samples received, five plasma samples scored a 3 + or 4+, the remainder of samples were 2 + or below (Supplemental Figure S2). Of the five samples with visibly high hemolysis three were samples that were quantity not sufficient and the other two yielded the minimum amount of plasma to test.

#### Comparison of viral load results between mailed self-collected FSB and clinic-collected venipuncture blood

Table 5 reports the VL results for 1:7 diluted plasma from the mailed, self-collected MCTs compared to venipuncture plasma collected in clinic on the same day. Of 24 participants previously reported to be virologically unsuppressed 13 were virally suppressed at the time of specimen collection, resulting in only 11 individuals with a quantifiable VL. For the 13 PWOH none had detectable RNA in both venipuncture and MCT FSB VL testing. Twenty-five participants with HIV on ART were virally suppressed in venipuncture samples. Of these, 17 were undetectable in both the venipuncture and matched self-collected MCT samples. One undetectable venipuncture sample resulted in detected but below the limit of quantification in the matched MCT. The remaining eight venipuncture samples had HIV detectable at < 30 copies/mL; five of these eight also had detectable HIV below the limit of quantitation in their MCT specimens, and the remaining three MCTs had no virus detected.

Venipuncture specimens from 11 participants with HIV had quantifiable virus, ranging from 1.9 to 6.2 log_10_ copies/mL (VL mean = 3.98; median = 3.70; interquartile range = 2.88 log_10_). One venipuncture plasma had quantifiable virus below the 2.3 log_10_ U = U benchmark (1.9 log_10_, 82 copies/mL); the matched FSB sample resulted in HIV also detected but below the quantification benchmark. All venipuncture VLs exceeding 2.3 log_10_ also resulted in quantifiable virus above the benchmark for the matched self-collected FSB. One high venipuncture VL (457,000 copies/mL) was 0.59 log_10_ higher that the matched MCT sample (118,000 copies/mL), slightly outside the expected 0.5 log_10_ variance. This did not practically affect the interpretation, because both results were of an elevated VL well in excess of the U = U benchmark.

**Fig. 5:**
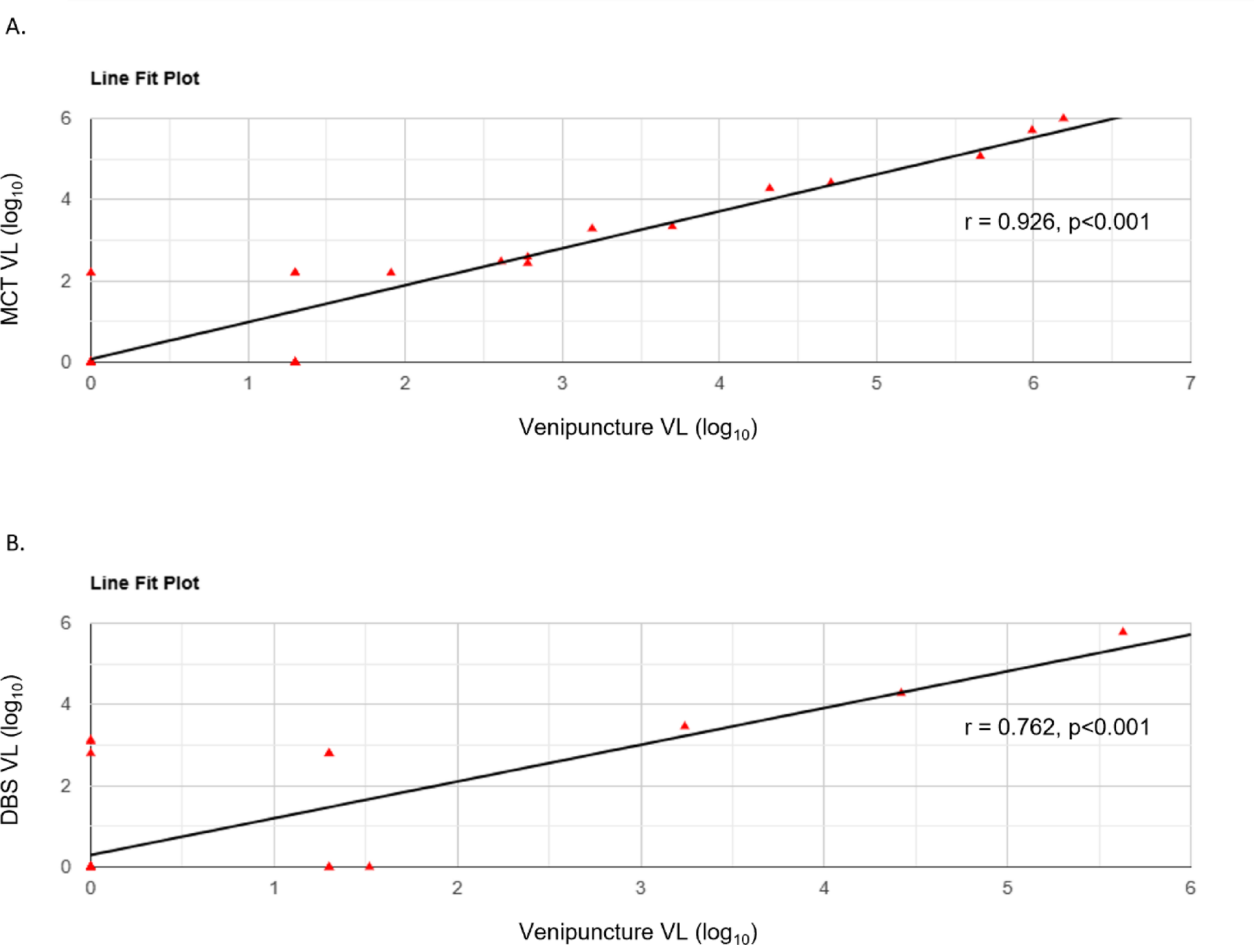
Results of the Pearson correlations between all viral load results from clinic-drawn venipuncture blood and self-collected microtainers (MCT) (A.) and dried blood spots (DBS) (B.). Viral load results below the limit of quantification for each sample type were assigned a numerical value of 1.3 log_10_ for venipuncture samples and 2.2 log_10_ for MCT or 2.8 log_10_ for DBS samples

The Pearson coefficient showed essentially perfect correlation between self-collected MCT results as compared to matched plasma results with respect to viral suppression above or below the 2.3 log_10_ U = U benchmark (*r* = 0.999, *p* < 0.001). Additionally, when taking into consideration all viral load values as an indication of quantitative similarity irrespective of the suppression benchmark the correlation remained very strong (*r* = 0.926, *p* < 0.001) (Fig. 5). In interpreting whether or not virus concentrations met the U = U prevention goal the results from self-collected MCT compared to venipuncture plasma were in perfect agreement (K = 1, SE = 0, 95% CI 1.0, 1.0).

**Table 5:**
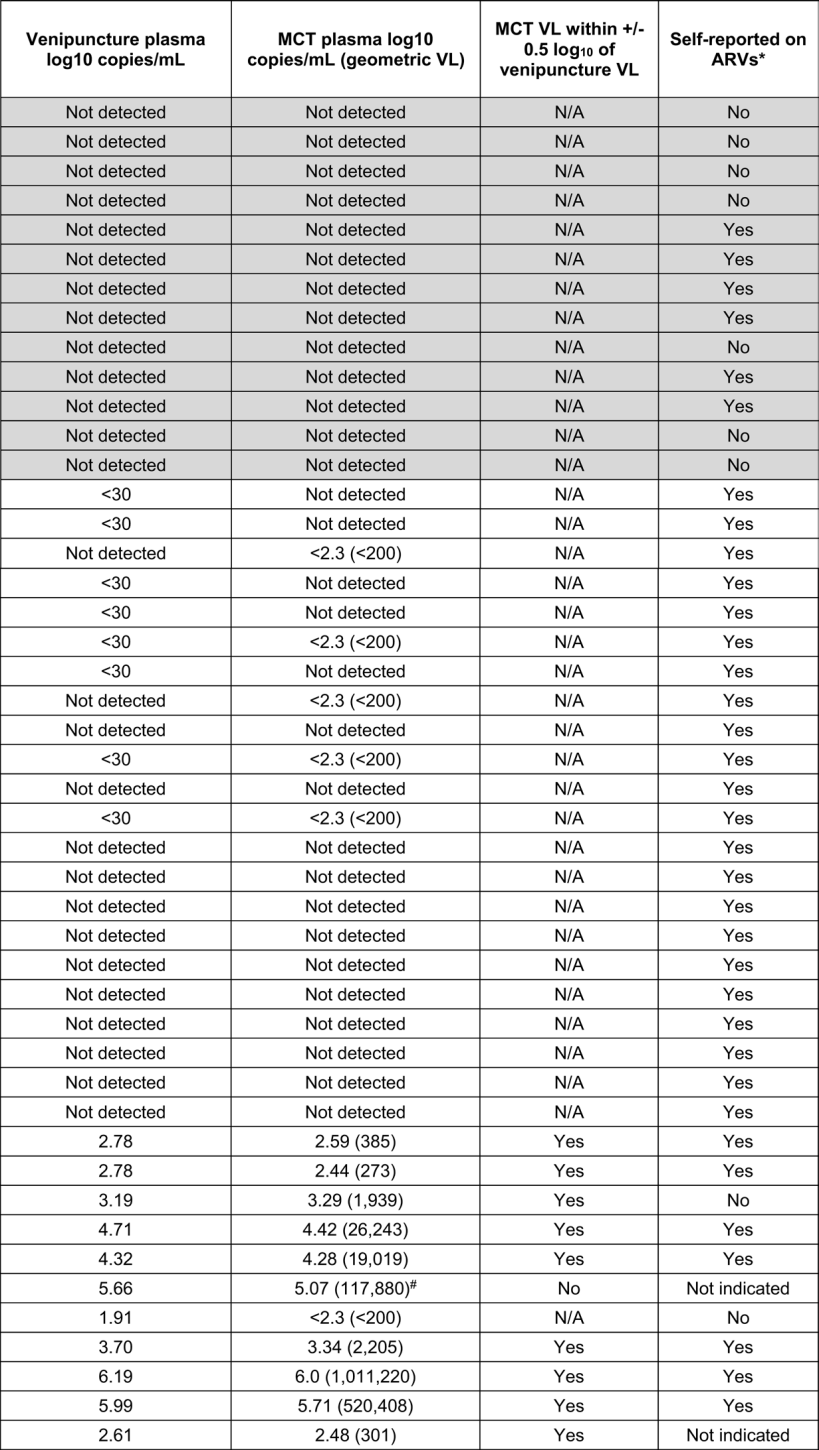
Viral load results for mailed self-collected microtainers (MCT) and matched clinic-collected venipuncture blood

Shaded rows indicate persons without HIV (PWOH); ^*^ reported PrEP for PWOH or ART for persons with HIV; N/A, not applicable; ^**#**^ result concordant with elevated VL but value was 0.59 log_10_ lower than venipuncture VL

#### Comparison of viral load results between mailed self-collected DBS and clinic-collected venipuncture blood

Venipuncture specimens from the second clinic visits were matched to same-day self-collected DBS. In this analysis 34/47 (72%) of DBS with matched venipuncture specimens were accepted for testing. The results from testing of 11 DBS from PWOH all matched the venipuncture results (no HIV detected; Table 6). For 15 PWH who had no detectable virus in their venipuncture sample, 12 also had no detectable virus in their DBS specimens, one was detectable but below quantification, and two were detectable and quantitated at 1,230 and 1,349 copies/mL, respectively. For four persons with quantifiable HIV in their venipuncture sample who had a matched DBS, three were also quantifiable in their DBS sample. The remaining DBS specimen that was below quantitation had a matched venipuncture VL of 33 copies/mL. The Pearson’s correlation between self-collected DBS and matched plasma with respect to viral suppression was significant (*r* = 0.762, *p* < 0.001) (Fig. 5); however, the kappa statistic only showed moderate agreement (K = 0.478).

**Table 6:**
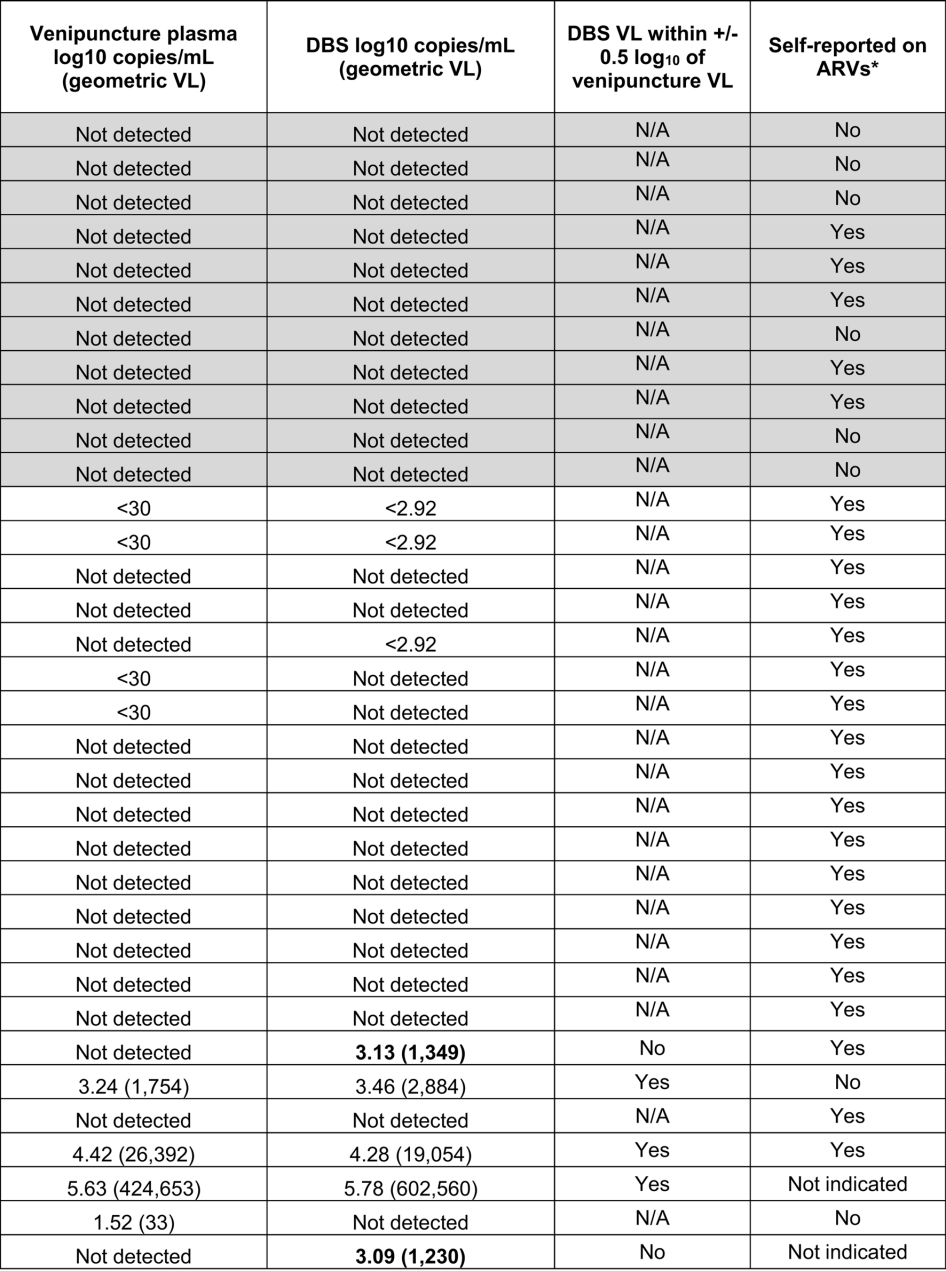
Viral load results for self-collected DBS and matched clinic-collected venipuncture specimens

Shaded rows indicate persons without HIV (PWOH); ^*^ reported PrEP for PWOH or ART for persons with HIV; N/A, not applicable; the bold DBS values are discordant with the venipuncture VL results

## Discussion

We performed an evaluation of a self-collected fingerstick blood protocol for remote viral suppression monitoring in direct comparison to clinic-collected venipuncture samples. Providing a semi-quantitative HIV RNA detection method for mail-in specimens would allow individuals living with HIV who face significant barriers to HIV care a means to monitor their viral suppression status. To reach the Ending the HIV Epidemic viral suppression goals we aimed to create an HIV RNA test procedure on self-collected specimens that supported the U = U suppression benchmark of 200 copies/mL. We established for this protocol that fingerstick blood-derived plasma quantitated at ≥ 210 copies/mL indicates VLs that are above the U = U prevention benchmark. We recognize that the precision of these results are subject to known variability in VL testing.

The laboratory acceptance criteria for mailed specimens were established on multiple time and temperature conditions to simulate plausible temperature ranges to which specimens may be exposed during shipment to the testing laboratory. These simulations suggested an acceptable upper tolerance of 36 °C for four days in transit and was applied in the field evaluation for all self-collected MCTs received from participants. It was not expected that the constant elevated temperatures applied in the analytical bench evaluations would be experienced during the entire four days of specimen transit; however, we assigned the bounds of acceptability to this worst-case condition for the purposes of this assessment. From the extended environmental stress testing that was performed, we found that shorter exposure times permitted higher temperature tolerances without degrading viral loads. At the upper tolerances tested, constant temperatures of up to 46 °C for up to eight hours did not impact specimen results. This suggested even in very harsh conditions same-day specimen courier services could support VL monitoring. Additional evaluation would be needed for other anticipated extreme conditions, including freezing temperatures.

Self-collected MCT results were highly concordant with FDA-approved conventional VL testing. Even venipuncture VLs which marginally exceeded the U = U benchmark (> 250 copies/mL) were quantifiable in the matched MCT specimens sent by participants. This key finding documents that self-collected specimens can accurately reflect the presence of virus above the biologically-relevant suppression target. However, there is an inherent weakness in applying a cutoff at an exact value because of the inter-run variability of nucleic acid testing. Such variability can lead to specimens with viral load concentrations around the threshold yielding results that might appear above or below the benchmark metric. For this reason, we have previously suggested a lower LOQ of 400 copies/mL for the dilution protocol with same-day specimen processing [8] when the aim is maximizing sensitivity for a quantitative test. Nevertheless, during the analytical evaluations, specimens that experienced environmental conditions that slightly exceeded established tolerances resulted in only a ≤ 0.5 log_10_ difference as compared to baseline MCT results. If these results were to be used for clinical care, providers would need to evaluate the aims of clinical management and might wish to order additional testing with venipuncture specimens if the uncertainty of the low-level concentration is relevant to clinical decision making. Even in such clinical settings, however, most MCT results would be sufficient for clinical decision making without the need for follow-up venipuncture-based testing.

The performance of the MCT protocol at higher VLs reflected excellent concordance with venipuncture values, but these findings were not pertinent to the suppression monitoring use-case described here. Nevertheless, these observations suggest that a quantitative assay using self-collected specimens might be feasible. Importantly, lysed HIV DNA-containing cells diluted in whole blood did not result in detectable HIV with the HIV-1 Quant DX assay as this test employs transcription-mediated amplification (TMA) to minimize amplification of DNA. The absence of an artificially inflated virus concentration with hemolyzed samples alleviates concern if cell lysis were to occur during fingerstick self-collection. Additionally, no PWH who was fully virally suppressed in their plasma specimen had quantifiable levels of HIV in their self-collected MCT specimens.

The DBS specimens were largely concordant with matched venipuncture results; however, DBS testing was not able to discern VL values below ~ 800 copies/mL. Moreover, for two (10%) participants with HIV who were suppressed to undetectable levels in their venipuncture plasma, their DBS result indicated a VL above 1000 copies/mL. This is a shortcoming of using a specimen type for which proviral DNA may artificially inflate an HIV RNA level if used on a test platform that will detect residual DNA. These limitations were reflected by the Kappa statistic resulting in only a moderate agreement between DBS and venipuncture samples in measuring benchmark suppression.

Sample size was a primary limitation to our analysis. An unexpected but favorable health outcome was that most previously unsuppressed persons were virally suppressed when they enrolled in the current study. This higher-than-expected level of suppression among people who self-reported lack of suppression together with the difficulty in recruiting PWH with unsuppressed viral loads limited our ability to reach our anticipated number of PWH for quantitative comparisons. This highlights an underlying difficulty in engaging persons who face social and structural barriers to HIV care; we hope these barriers could be overcome if options for remote care become available. Several participants initially found it difficult to obtain FSB; however, ~ 80% of persons who had experienced difficulty with collection opted to try again and were able to collect a suitable specimen. As with any HIV prevention tool, not all persons are accepting of every test collection method that may be available. Yet with additional experience it can be envisioned that acceptance of FSB collection could increase. Additionally, other capillary blood collection devices could be employed as a reflex option to people who experience difficulty using traditional lancets. Such a reflex approach to offering more expensive but more acceptable sampling devices would allow greater opportunities for self-testing. However, the higher costs may be a deterrent to testing and, therefore, needs careful consideration.

Remote viral suppression monitoring is an opportunity to increase access to additional HIV care services and promote expansion of HIV telemedicine for the care of PWH. For mailed specimens that have quantifiable HIV-1 RNA, additional VL testing on venipuncture specimens may be appropriate for clinical management of persons on antiretroviral therapy. It must be appreciated, however, that for some individuals in-person clinic follow-up may not be feasible. Expanded access to self-collection testing opportunities and tele-counseling to address any difficulties experienced with maintaining treatment adherence will open new paths to convenient and routine viral load monitoring. Importantly, cost-conscious, autonomous access to discrete testing will be vital for improving HIV suppression rates for many PWH who face barriers to the continuum of HIV care. Self-collected specimens hold promise for specific clinical uses, and self-directed remote HIV testing desired by the community can be empowering for individuals.

## Supplementary Information

Below is the link to the electronic supplementary material.

Supplementary Material 1

## Data Availability

Data is provided within the manuscript or supplementary information files.
