# Supplementary material for: A cross-sectional analysis of fingerstick blood self-microcollection for remote HIV suppression monitoring in Atlanta, Georgia, USA: a path to expanding access to continuum of care
Source: AIDS Res Ther. 2025 Aug 30;22:87. doi: 10.1186/s12981-025-00781-1 (PMC12398179; doi:10.1186/s12981-025-00781-1)
Supplement: Supplementary file 1 — Supplementary Material 1 [file 12981_2025_781_MOESM1_ESM.docx]

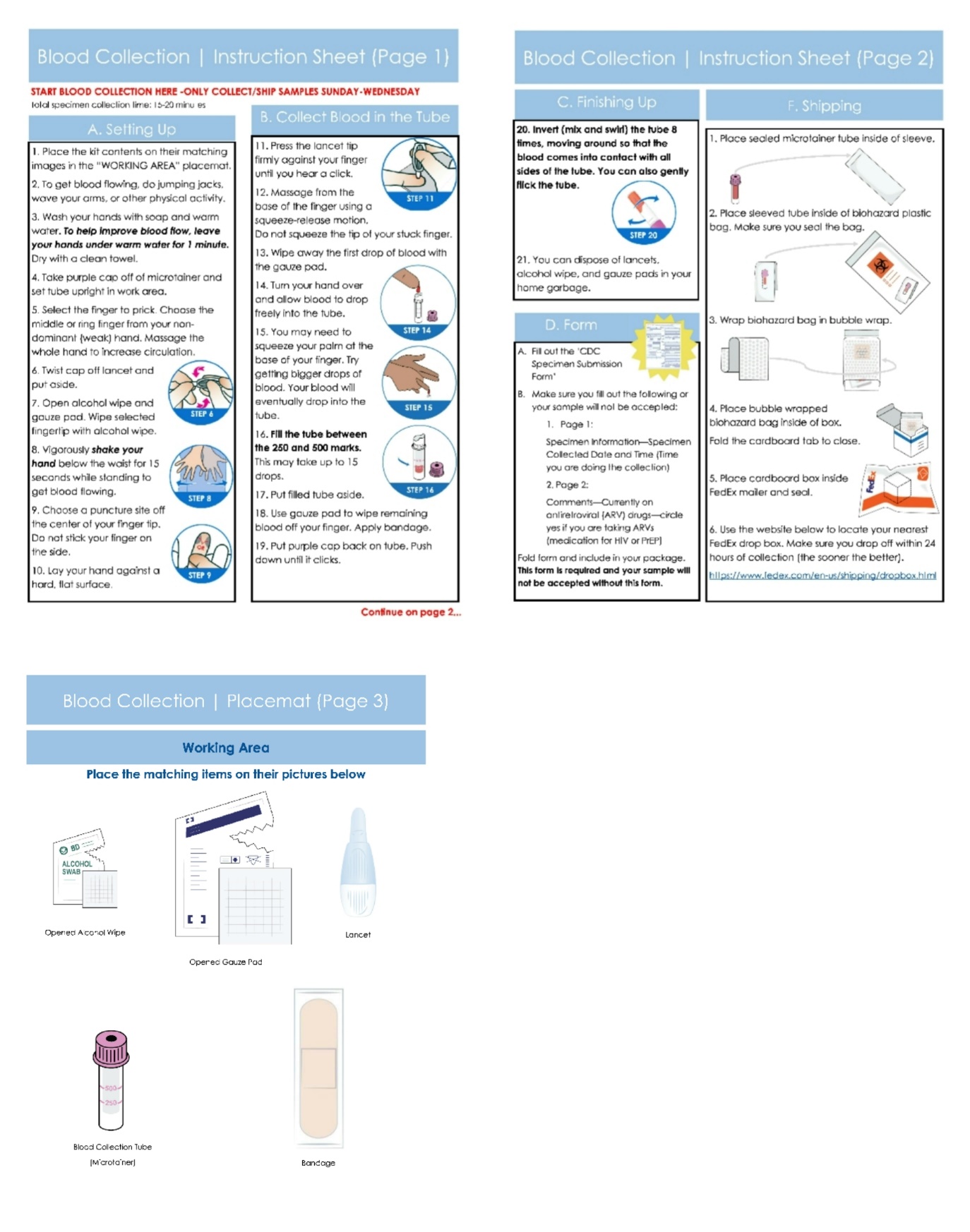


Figure S1. Instructions to participants included in the mailed self-collection kit.


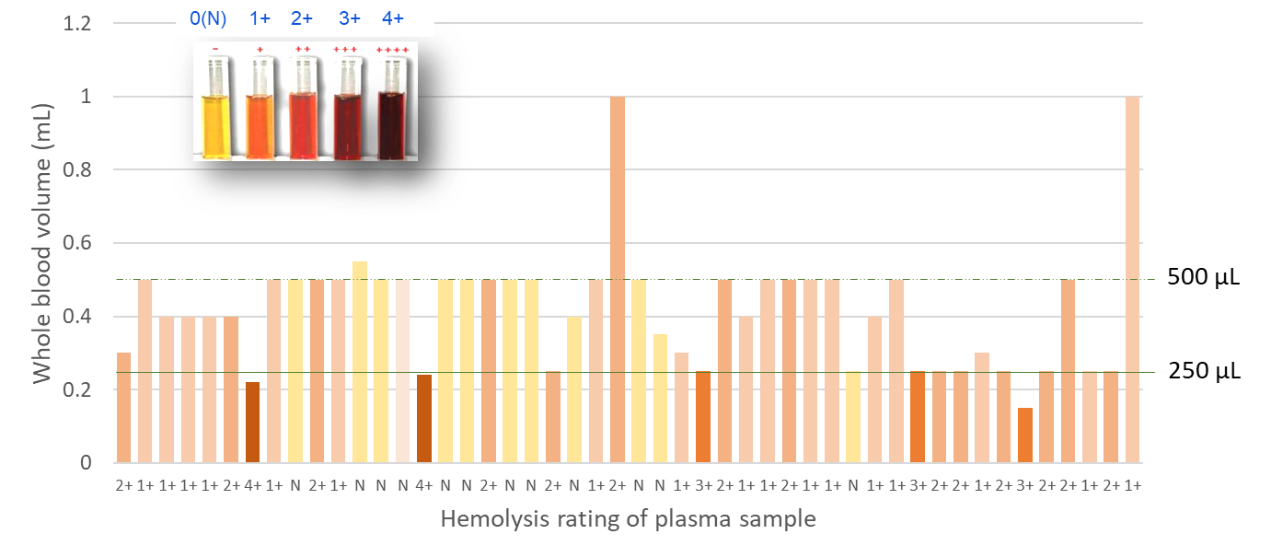


Figure S2. Hemolysis index for visually grading the level of hemolysis of plasma prepared from participant-mailed Microtainer fingerstick blood. Indices of 3 + and 4 + would assume blood lysis due to difficulty in obtaining samples. This assumption did correlate with unacceptably low sample volumes.
